# Supplementary material for: Memory strength gates the involvement of a CREB-dependent cortical fear engram in remote memory
Source: Nat Commun. 2019 May 24;10:2315. doi: 10.1038/s41467-019-10266-1 (PMC6534583; doi:10.1038/s41467-019-10266-1)
Supplement: Supplementary file 1 — Supplementary Information [file 41467_2019_10266_MOESM1_ESM.pdf]

Supplementary Information

Memory strength gates the involvement of a CREB-dependent cortical fear engram in remote memory

Matos et al.

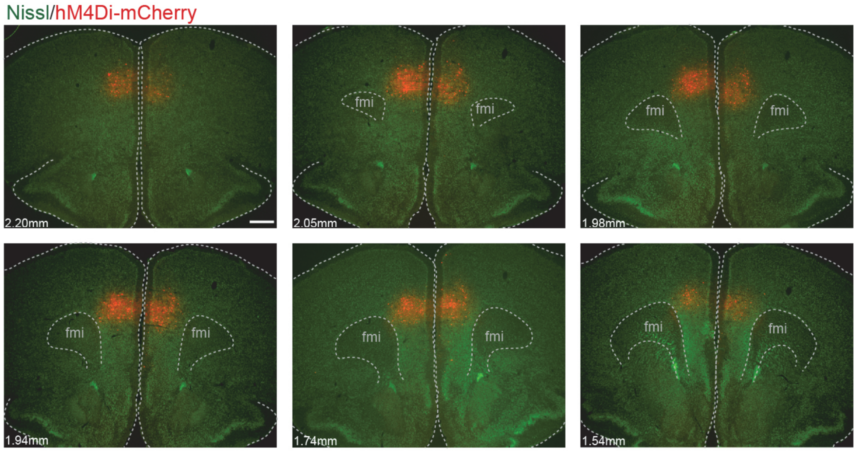

Supplementary Figure 1 Representative example of hM4Di-mCherry expression in the mPFC after CFC and 4TM treatment. A mixture of AAV-Fos::CreER^{T2} and AAV-hSyn::DIO-hM4Di-mCherry was injected into the dorsal region of the mPFC. Tagged neurons expressing hM4Di-mCherry (red) were observed along the entire rostro-caudal axis of the dorsal mPFC. In the bottom right of each image, coordinates relative to Bregma are shown based on the Paxinos and Watson mouse brain atlas. Scale bar = 500 μm. fmi = forceps minor of the corpus callosum.

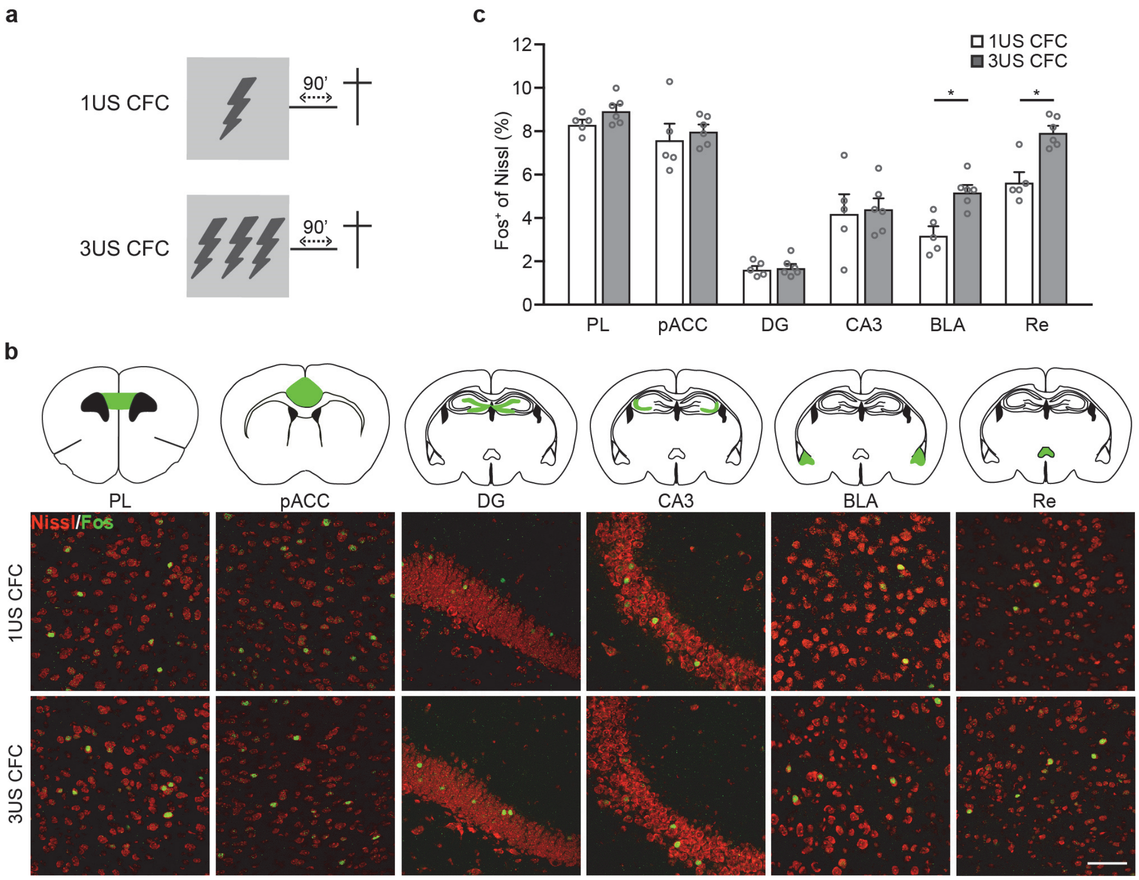

Supplementary Figure 2 Fos expression after 1US and 3US CFC. **a** Left: experimental design. 1US CFC (n = 5 mice); 3US CFC (n = 6 mice). **b** Top: illustration of a coronal brain section indicating the region (green) where Fos^+ neurons were analyzed. Bottom: representative examples of $Nissl^+$ (red) and Fos^+ cells (green). Scale bar = 50 μm. **c** Percentage of Fos^+ cells in each region. Unpaired *t*-test: BLA $t_9 = 4.164$, $^*p = 0.0024$; Re $t_9 = 4.526$, $^*p = 0.0014$. All bar graphs show means + s.e.m. Source data are provided as a Source Data file.

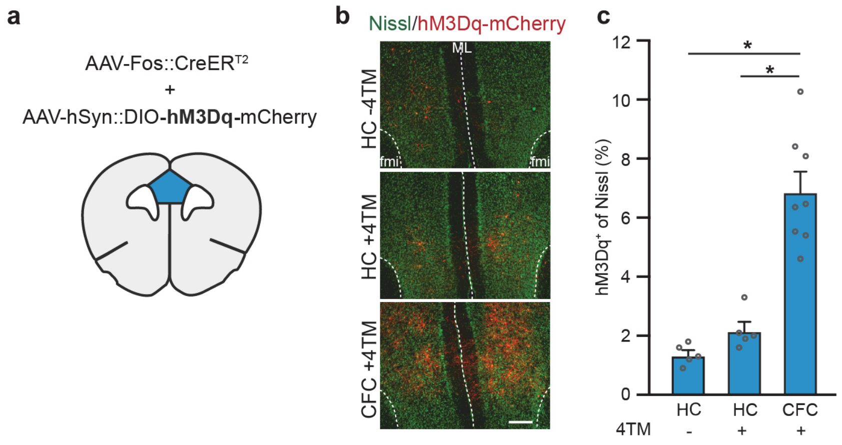

Supplementary Figure 3 Dual virus TRAP enables inducible activity-dependent tagging of mPFC neurons with hM3Dq-mCherry. **a** A mixture of AAV-Fos::CreER^{T2} and AAV-hSyn::DIO-hM3Dq-mCherry was bilaterally infused into the mPFC. **b** Expression of hM3Dq-mCherry (red) in mPFC in home-cage (HC) control mice without 4TM treatment (HC -4TM; n = 5) and with 4TM treatment (HC +4TM; n = 5), and in mice that underwent contextual fear conditioning followed by 4TM treatment (CFC +4TM; n = 8). fmi = forceps minor of the corpus callosum. ML = midline. Left: scale bar = 250 µm. **c** Percentage of hM3Dq-mCherry^{+} cells in mPFC. One-way ANOVA: $F_{(2,15)}$=32.79, $p < 0.0001$; post-hoc Bonferroni test: CFC *vs*. HC -4TM, *$p <$ 0.0001, CFC *vs*. HC +4TM, *$p < 0.0001$. Bar graph shows mean + s.e.m. Source data are provided as a Source Data file.

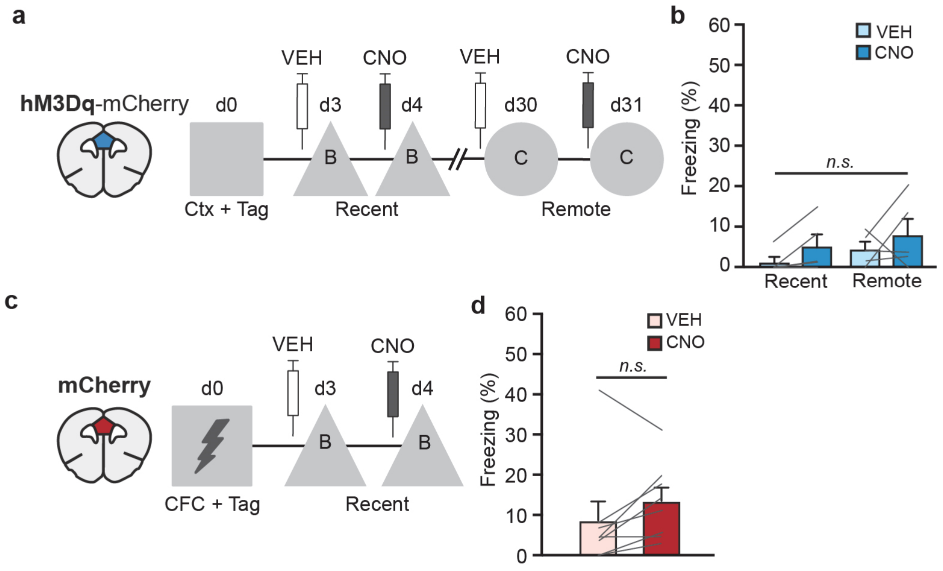

Supplementary Figure 4 Stimulation of mPFC neurons in control mice did not enhance freezing. **a** Experimental design. On d0, neurons activated during exploration of a neutral context in the absence of a foot-shock were tagged with hM3Dq-mCherry. Changes in freezing levels were assessed during vehicle (VEH) or CNO sessions in either context B (recent) or Context C (remote). **b** No changes were observed after CNO administration when compared to VEH at both time points. Repeated measures ANOVA: $F_{(1,4)} = 2.117$, $p = 0.184$ (n = 5 mice). Note that not all individual data points are visible as several animals did not show freezing behavior. **c** Neurons activated during CFC were tagged with mCherry. Freezing levels were assessed after VEH and CNO treatment in context B. **d** CNO did not enhance freezing compared with VEH. Paired *t*-test, $t_7 = 1.786$, $p = 0.117$ (n = 8 mice). Bar graphs show mean + s.e.m. Source data are provided as a Source Data file.

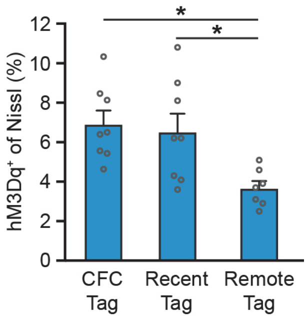

Supplementary Figure 5 Number of hM3Dq+ neurons in CFC- and retrieval-tagged experimental groups. 'CFC Tag' reflects the group presented in Fig. 4a, b (n = 8 mice). 'Recent Tag' reflects the group presented in Fig. 5a, b (n = 8 mice). 'Remote Tag' reflects the group presented in Fig. 5c, d (n = 7 mice). One-way ANOVA $F_{(2,20)}= 5.95$, $p = 0.009$. Post-hoc Bonferroni test: CFC tag *vs*. Remote tag *$p = 0.014$; Recent tag *vs*. Remote tag *$p = 0.033$. Bar graph shows means + s.e.m. Source data are provided as a Source Data file.

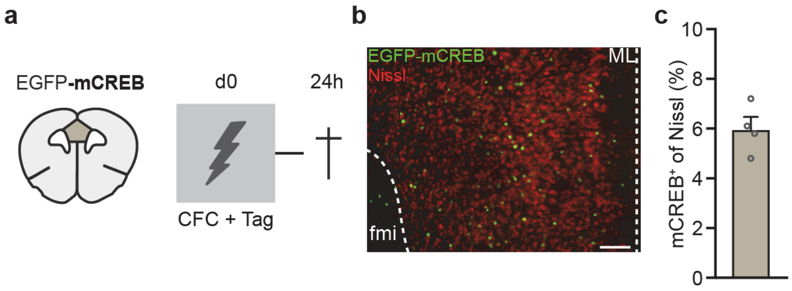

Supplementary Figure 6 EGFP-mCREB was detectable 24 h after CFC. **a** Neurons activated during CFC were tagged with EGFP-mCREB and mice were perfused 24 h later. **b** Representative image of EGFP-mCREB$^+$ neurons in mPFC. Scale bar = 100 µm. fmi = forceps minor of the corpus callosum. ML = midline. **c** Percentage of mCREB$^+$ neurons 24 h after CFC (n = 4 mice). Bar graph shows mean + s.e.m. Source data are provided as a Source Data file.

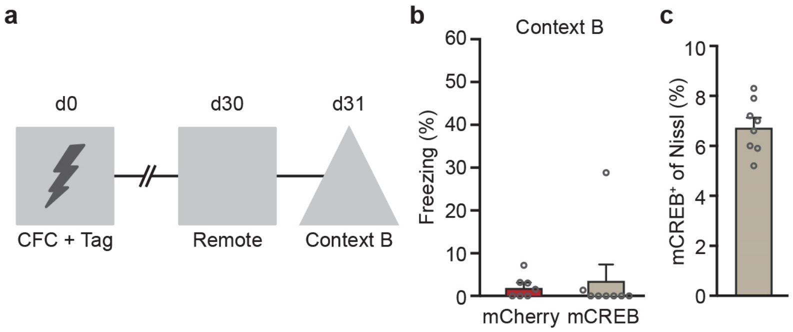

Supplementary Figure 7 EGFP-mCREB expression in CFC-tagged neurons did not induce expression of generalized fear. **a** Experimental outline. mPFC neurons activated during CFC were tagged with EGFP-mCREB and remote memory was first assessed on day 30 in the CFC context (see Figure 6) and subsequently on day 31 in a neutral context B. **b** Both groups showed similar, very low levels of freezing in context B. Mann-Whitney U test: U = 19, *p* = 0.276. **c** Percentage of CFC-tagged mPFC neurons expressing EGFP-mCREB. Bar graphs show mean + s.e.m. Source data are provided as a Source Data file.

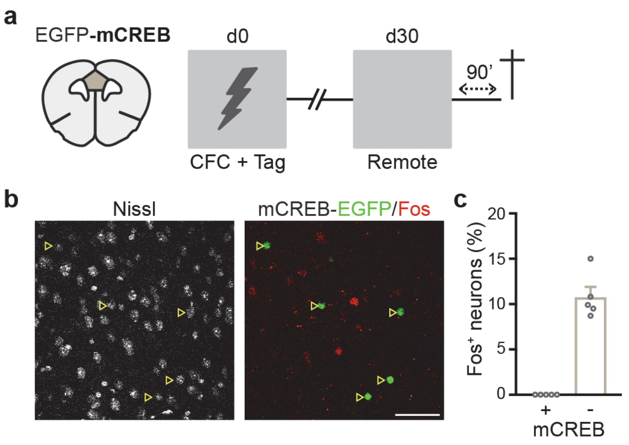

Supplementary Figure 8 Remote memory retrieval did not induce Fos expression in mCREB$^+$ neurons. **a** Experimental design. Neurons activated during CFC were tagged with EGFP-mCREB and mice were subsequently perfused after a re-exposure to the conditioning context on day 30 after training. **b** Example of mCREB$^+$ and Fos$^+$ cells within the total population of mPFC neurons (Nissl$^+$). Yellow outlined arrowheads indicate mCREB$^+$/Fos$^-$ cells. **c** Percentage of Fos$^+$ cells within the mCREB$^+$ and mCREB$^-$ population. Bar graph shows mean + s.e.m. Source data are provided as a Source Data file.

a

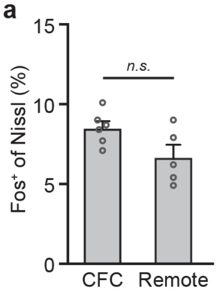

Supplementary Figure 9 The percentage of Fos$^+$ neurons did not significantly differ after CFC and remote memory retrieval. Fos expression was analyzed in mice that were perfused after 1US CFC or after re-exposure to the conditioning context at day 30 following 1US CFC. Data of the CFC group was copied from Figure 1c. Data of the remote group was derived from the reactivation experiment shown in Figure 4f-h. *n.s.* = not significant. Bar graph shows mean + s.e.m. Source data are provided as a Source Data file.
