# Supplementary material for: Memory strength gates the involvement of a CREB-dependent cortical fear engram in remote memory
Source: Nat Commun. 2019 May 24;10:2315. doi: 10.1038/s41467-019-10266-1 (PMC6534583; doi:10.1038/s41467-019-10266-1)
Supplement: Supplementary file 3 — Reporting Summary [file 41467_2019_10266_MOESM3_ESM.pdf]

nature research

Corresponding author(s): M.C. van den Oever

Reporting Summary

Nature Research wishes to improve the reproducibility of the work that we publish. This form provides structure for consistency and transparency in reporting. For further information on Nature Research policies, see Authors & Referees and the Editorial Policy Checklist.

Statistical parameters

When statistical analyses are reported, confirm that the following items are present in the relevant location (e.g. figure legend, table legend, main text, or Methods section).

n/a	Confirmed	
☐	☒	The exact sample size (n) for each experimental group/condition, given as a discrete number and unit of measurement
☐	☒	An indication of whether measurements were taken from distinct samples or whether the same sample was measured repeatedly
☐	☒	The statistical test(s) used AND whether they are one- or two-sided<br>*Only common tests should be described solely by name; describe more complex techniques in the Methods section.*
☒	☐	A description of all covariates tested
☐	☒	A description of any assumptions or corrections, such as tests of normality and adjustment for multiple comparisons
☐	☒	A full description of the statistics including central tendency (e.g. means) or other basic estimates (e.g. regression coefficient) AND variation (e.g. standard deviation) or associated estimates of uncertainty (e.g. confidence intervals)
☐	☒	For null hypothesis testing, the test statistic (e.g. F, t, r) with confidence intervals, effect sizes, degrees of freedom and P value noted<br>*Give P values as exact values whenever suitable.*
☒	☐	For Bayesian analysis, information on the choice of priors and Markov chain Monte Carlo settings
☒	☐	For hierarchical and complex designs, identification of the appropriate level for tests and full reporting of outcomes
☒	☐	Estimates of effect sizes (e.g. Cohen's d, Pearson's r), indicating how they were calculated
☐	☒	Clearly defined error bars<br>*State explicitly what error bars represent (e.g. SD, SE, CI)*

Our web collection on statistics for biologists may be useful.

Software and code

Policy information about availability of computer code

Data collection	Ethovision XT (Noldus), LSM510 (Zeiss), pClamp (Molecular Devices)
Data analysis	Excel (Microsoft), SPSS (IBM), Image J (NIH), MATLAB (Mathworks), Prism (Graphpad)

For manuscripts utilizing custom algorithms or software that are central to the research but not yet described in published literature, software must be made available to editors/reviewers upon request. We strongly encourage code deposition in a community repository (e.g. GitHub). See the Nature Research guidelines for submitting code & software for further information.

Data

Policy information about availability of data

All manuscripts must include a data availability statement. This statement should provide the following information, where applicable:

- Accession codes, unique identifiers, or web links for publicly available datasets
- A list of figures that have associated raw data
- A description of any restrictions on data availability

The datasets generated during and/or analyzed during the current study are available from the corresponding author on reasonable request.

Field-specific reporting

Please select the best fit for your research. If you are not sure, read the appropriate sections before making your selection.

☒ Life sciences ☐ Behavioural & social sciences ☐ Ecological, evolutionary & environmental sciences

For a reference copy of the document with all sections, see nature.com/authors/policies/ReportingSummary-flat.pdf

Life sciences study design

All studies must disclose on these points even when the disclosure is negative.

Sample size	Initial sample sizes were determined based on biological variance observed in previous fear conditioning experiments and immunohistochemical quantifications. Some animals had to be excluded from the analyses (see below). Final sample sizes are indicated in the figure legends.
Data exclusions	Virus misplacements was a priori set as an exclusion criterion. The number of excluded mice based on virus misplacements are described in the Methods (Statistical analysis). In addition, one mouse of the '1US CFC' group in supplementary figure 2 was excluded due to tissue damage.
Replication	Critical experiments with a statistically significant outcome were replicated successfully.
Randomization	For each experiment, mice were age and weight-matched and randomly assigned to groups
Blinding	Blinding was not necessary for fear conditioning experiments as freezing behavior was automatically scored using constant settings in Ethovision (Noldus). For imaging and histological analyses, experimenters were blinded to group allocation of individual animals.

Reporting for specific materials, systems and methods

Materials & experimental systems

n/a	Involved in the study
☐	☒ Unique biological materials
☐	☒ Antibodies
☒	☐ Eukaryotic cell lines
☒	☐ Palaeontology
☐	☒ Animals and other organisms
☒	☐ Human research participants

Methods

n/a	Involved in the study
☒	☐ ChIP-seq
☒	☐ Flow cytometry
☒	☐ MRI-based neuroimaging

Unique biological materials

Policy information about availability of materials

Obtaining unique materials	Unique materials (AAV constructs) are available from the corresponding author.

Antibodies

Antibodies used	rabbit anti-Fos (1:500, sc52, Santa Cruz, USA), rabbit anti-RFP (1:1000, 600-401-379S, Rockland, USA)
Validation	The antibodies are commercially available and successfully applied in previous studies (e.g. see Ramirez et al., Science 2013). Tissue stained without primary antibody served to rule out the possibility of non-specific binding of secondary antibody to the tissue. RFP: Statement of manufacturer - Assay by immunoelectrophoresis resulted in a single precipitin arc against anti-Rabbit Serum and purified and partially purified Red Fluorescent Protein (Discosoma). No reaction was observed against Human, Mouse or Rat serum proteins.

Animals and other organisms

Policy information about studies involving animals; ARRIVE guidelines recommended for reporting animal research

Laboratory animals	Mice, C57Bl/6J, males, 2-3 months old at the start of experiments
Wild animals	*Provide details on animals observed in or captured in the field; report species, sex and age where possible. Describe how animals were caught and transported and what happened to captive animals after the study (if killed, explain why and describe method; if released, say where and when) OR state that the study did not involve wild animals.*
Field-collected samples	*For laboratory work with field-collected samples, describe all relevant parameters such as housing, maintenance, temperature, photoperiod and end-of-experiment protocol OR state that the study did not involve samples collected from the field.*

April 2018
